# Regulation of fiber-specific actin expression by the *Drosophila* SRF ortholog Blistered

**DOI:** 10.1242/dev.164129

**Published:** 2019-04-04

**Authors:** Ashley A. DeAguero, Lizzet Castillo, Sandy T. Oas, Kaveh Kiani, Anton L. Bryantsev, Richard M. Cripps

**Affiliations:** 1 Department of Biology, University of New Mexico, Albuquerque, NM 87131, USA; 2 Department of Biology, San Diego State University, San Diego, CA 92182, USA; 3 Department of Molecular and Cellular Biology, Kennesaw State University, Kennesaw, GA 30144, USA

**Keywords:** *Drosophila*, Flight muscle, Serum response factor, SRF, Actin

## Abstract

Serum response factor (SRF) has an established role in controlling actin homeostasis in mammalian cells, yet its role in non-vertebrate muscle development has remained enigmatic. Here, we demonstrate that the single *Drosophila* SRF ortholog, termed Blistered (Bs), is expressed in all adult muscles, but Bs is required for muscle organization only in the adult indirect flight muscles. Bs is a direct activator of the flight muscle actin gene *Act88F*, via a conserved promoter-proximal binding site. However, Bs only activates *Act88F* expression in the context of the flight muscle regulatory program provided by the Pbx and Meis orthologs Extradenticle and Homothorax, and appears to function in a similar manner to mammalian SRF in muscle maturation. These studies place Bs in a regulatory framework where it functions to sustain the flight muscle phenotype in *Drosophila*. Our studies uncover an evolutionarily ancient role for SRF in regulating muscle actin expression, and provide a model for how SRF might function to sustain muscle fate downstream of pioneer factors.

## Abstract (precis)

**Summary:** *Drosophila* Serum response factor has a fiber-specific role in controlling actin expression in the adult flight muscles, and functions downstream of the putative pioneer factors Extradenticle and Homothorax.

## INTRODUCTION

Muscle tissue has diversified into specialized types to accommodate a spectrum of functional demands. In mammals, smooth muscles mediate continuous tonic contractions, cardiac and slow-twitch skeletal fibers are responsible for intermittent persistent contractions, and fast-twitch skeletal fibers generate powerful but transient contractions (Squire, 1986). This muscle heterogeneity is reflected by selective expression of distinct structural genes, including actin genes. Smooth, cardiac and skeletal muscles express their own actin isoforms that contribute to the differences in muscle contractile parameters (Perrin and Ervasti, 2010).

Understanding the regulation of actin gene expression has become a paradigm for understanding the transcriptional signatures that promote muscle differentiation and diversification. One transcription factor known to regulate actin expression is Serum response factor (SRF). First identified as a regulator of FOS expression (Norman et al., 1988), SRF plays a major role in muscle differentiation in mammals, where it directly regulates actin gene expression, among many others, through interaction with a conserved DNA-binding motif termed the CArG box (reviewed by Miano et al., 2007).

*In vivo* experiments underline this role for SRF in mammalian muscle differentiation: while SRF is required for mesoderm specification early during embryogenesis in mice (Arsenian et al., 1998), conditional knockouts revealed requirements for SRF in cardiac development and function (Parlakian et al., 2005, 2004). Moreover, loss of SRF function during later embryonic development resulted in perinatal lethality arising from a failure of muscle growth following initial specification (Li et al., 2005). In most cases, there was a reduction in the expression of genes encoding contractile proteins, in particular of the muscle actin genes (Li et al., 2005; Parlakian et al., 2005).

Given that many animal genomes have an SRF gene, researchers have sought to define the evolutionary depth of the involvement of SRF in myogenesis. While the *Drosophila* genome contains a single SRF ortholog, termed *blistered* (*bs*), that is expressed in embryonic muscles, *bs* mutants do not show obvious muscle defects. Instead, *bs* is necessary for normal tracheal development and viability (Affolter et al., 1994; Fristrom et al., 1994). In *C. elegans*, the SRF ortholog UNC-120 can weakly activate the myogenic program in a blastomere conversion assay, and *unc-120* null mutants show muscle formation, although with progressive muscle weakness (Fukushige et al., 2006). In addition, UNC-120 appears to have overlapping functions with other myogenic factors, including the *C. elegans* orthologs of MyoD and Hand (Baugh et al., 2005). These studies suggest a more muted role for SRF orthologs in invertebrate muscle development.

In this study, we investigate the role of *bs* in the formation of the adult muscles of *Drosophila*. The adult thorax consists of two distinct skeletal muscle fiber types: the fibrillar indirect flight muscles (IFMs) that provide the power for flight; and tubular muscles that are required for posture and walking, including the tergal depressor of the trochanter (TDT or jump muscle), which is responsible for jumping. Recent studies have demonstrated that the flight muscles are specified by the Pbx1 and Meis1 orthologs Extradenticle and Homothorax, respectively (Bryantsev et al., 2012b), and by the zinc-finger transcription factor Spalt-major (Schönbauer et al., 2011).

We show that knockdown (KD) of *bs* expression during adult myogenesis reveals a requirement for *bs* function in controlling actin expression in the flight muscles, whereas the jump muscles retain a normal morphology. Bs activates expression of the flight muscle actin gene *Act88F* via a conserved proximal CArG box, but Bs can only promote *Act88F* expression in the context of flight muscle specification by Exd and Hth. These studies uncover a unique fiber-specific role for SRF in skeletal muscle differentiation. Moreover, our results parallel findings in mammals by showing that SRF is required for muscle maturation, and define a deep evolutionary role for SRF proteins in regulating muscle development.

## RESULTS

### Downregulation of *bs* during adult myogenesis only affects the indirect flight muscles

**Fig. 1.**
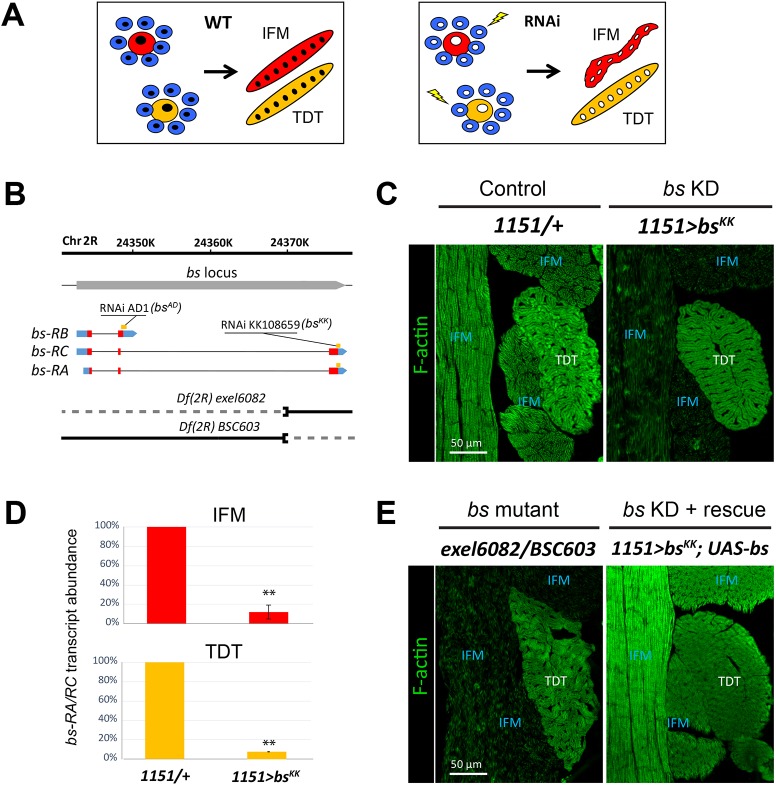
**Downregulation of *blistered* (*bs*) affects indirect fight muscle development.** (A) Schematics of RNAi screening for fiber-specific factors. Colored circles represent myoblasts (smaller size) and founder cells (larger size) that contain single nuclei (black circles). Multinucleate ovals represent IFM (red) and TDT (green), two muscles arising from different differentiation programs. Lightning bolts indicate RNAi-based knockdown (KD) of candidate genes within all myoblasts and founder cells (empty circles). Corrupted shape signifies affected muscle fiber phenotype. (B) *blistered* locus with chromosomal coordinates (top), gene boundary (gray box) and *bs* transcript isoforms (RA, RB and RC) with introns (thin lines) and exons (boxes) with coding (red) and noncoding (blue) regions. Orange bars show target sites for the RNAi constructs used in this study. On the bottom, two lines represent two genetic deletions with retained (solid line) and removed (dashed) genomic sequences. (C) Cryosectioned thoraces of control and *bs* knockdown (KD) flies with muscles stained for polymerized actin (F-actin, green). There is a strong, selective reduction in the green signal in IFMs upon *bs* KD. (D) qPCR-based quantification of *bs* transcripts in IFM and TDT muscles dissected from control and *bs* KD young adults. Data are mean±s.d. **P<0.01 (*t*-test). (E) Thoracic muscles in *bs* trans-heterozygous mutant (*exel6082/BSC603*) and genetically rescued *bs* KD flies. The restoration of F-actin staining in IFMs of the rescued fly (*1151>bs^KK^; UAS-bs*). IFM, indirect flight muscle; TDT, jump muscle.

We have been conducting a screen to identify transcription factors involved in *Drosophila* adult myogenesis (Bryantsev et al., 2012b). RNAi-based KD of transcription factor genes was induced in myoblasts and founder cells in developing pupae, and the phenotypes were assessed in mature muscles. Although all developing adult muscles were targeted, we anticipated some KDs might affect a subset of muscles, owing to the different myogenic programs of different adult muscle types (Fig. 1A). This was the case for the KD of the gene *blistered* (*bs*), which encodes *Drosophila* SRF (see below).

The *bs* gene is located on chromosome 2R, and encodes three annotated transcripts producing two different protein isoforms through alternative mRNA splicing (Fig. 1B). When we knocked down transcripts representing the RA and RC isoforms of *bs* using the RNAi line KK108659 (*bs^KK^*), the *bs* KD flies eclosed normally but were flightless.

Morphological analysis revealed that *bs* KD specifically affected the largest adult muscles: the indirect flight muscles (IFMs). Even though the IFMs were correctly specified, the flight muscles had a low polymerized actin content (Fig. 1C). Other somatic muscles, including the tergal depressor of the trochanter (TDT, or jump muscle), did not show any visible morphological defects (Fig. 1C), suggesting *bs* has a specific role in the flight muscles.

We confirmed that the phenotypes we observed were due to *bs* KD. First, we assessed *bs-RA/RC* transcript levels in flight and jump muscles using quantitative RT-PCR. We observed ∼90% reduction in *bs-RA/RC* transcript levels in both the flight and jump muscles of KDs (Fig. 1D), indicating that the KD does indeed affect *bs* expression levels.

Second, we determined whether mutants for *bs* also had flight muscle defects. Although *bs* null alleles are lethal (Affolter et al., 1994), a trans-heterozygous combination of two deletions overlapping the *bs* locus (shown diagrammatically in Fig. 1B) was viable, and showed a blistered wing phenotype characteristic of classical *bs* alleles (Bridges and Morgan, 1919). This combination enables normal expression of the *bs-RB* transcript, but deletes the 3′ coding exon that is characteristic of the *bs-RC* and *bs-RA* transcripts (Fig. 1B). The RNAi construct used for our screen (KK108659) also specifically targeted the *bs-RC* and *bs-RA* transcripts (Fig. 1B). Consistent with our RNAi results, the trans-heterozygous combination *Df(2R)exel6082/Df(2R)BSC603* showed the same adult muscle phenotype as the *bs* KD, where the flight muscles were affected but the jump muscles appeared normal (Fig. 1E, left panel).

Third, we carried out a genetic rescue experiment, where we expressed in the *bs^KK^* genetic KD background an HA-tagged *bs* isoform that is not targeted by the *bs^KK^* RNAi. While this genetic combination did not rescue flight, there was significant improvement in IFM myofibril formation in the rescue compared to the *bs* KD (Fig. 1E, right panel).

We conclude that *Drosophila* SRF shows a fiber-specific requirement in IFM differentiation.

### Myofibril defects in *bs* KD IFM

**Fig. 2.**
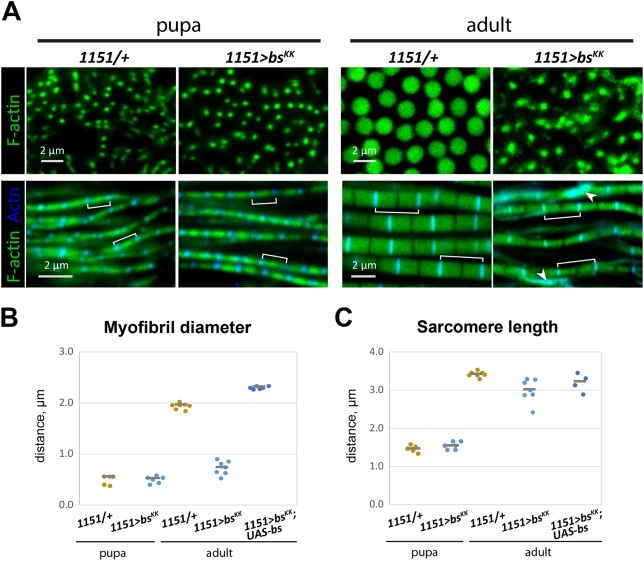
***bs* affects myofibril diameter in flight muscles.** (A) Cross-sectional (upper panels) and longitudinal (lower panels) views of IFM myofibrils in pupae (48 h apf) and adult flies, under control and *bs* knockdown (KD) conditions. Sarcomeres (brackets) are revealed by alpha-actinin immunostaining for Z-lines (blue) and counterstained using phalloidin for F-actin (green). Arrowheads show excess of alpha-actinin staining, concentrated outside the Z-lines. (B,C) Distribution of myofibril diameters (B) and sarcomere lengths (C) from 48 h apf pupae and adults under three genetic conditions: control (*1151*/+); *bs* KD (*1151*>*bs^KK^*) and genetic rescue of *bs* KD (*1151*>*bs^KK^*; *UAS-bs*). Median results from individual flies (20-100 myofibrils analyzed per fly) are plotted (four to seven per group). Gray horizontal lines show calculated median positions within each group.

To gain insight into the role of Bs in IFM myofibrillogenesis, we investigated the structural defects in *bs* KD and mutant animals. To determine whether this phenotype was a failure of muscle differentiation, we analyzed IFM myofibril structure in control and *bs* KD animals at 48 h after puparium formation (APF), approximately half-way through pupal development. We also stained these samples for accumulation of the Z-line protein α-actinin, to visualize sarcomere structure. All data are included in Table S1, and aggregate data shown are in Fig. 2. We found that control and *bs* KD myofibrils were similar to one another at 48 h APF (Fig. 2A, left panels), suggesting that the initial steps of myofibril formation were normal in the *bs* KD animals, although we note that we may not be able to observe subtle defects in the KDs. However, whereas controls myofibrils grew significantly during the latter half of pupal development, there was little growth in the *bs* KD myofibrils (Fig. 2B, right panels).

We quantified sarcomere length and myofibril diameter at these time-points. At 48 h APF, control and *bs* KD myofibrils had similar diameters of ∼0.5 µm. However, by the young adult stage the control myofibrils had grown to ∼2.0 µm in diameter, whereas those of the *bs* KD animals were largely unchanged. The failure of myofibril growth in the *bs* KD IFMs was rescued by expression of *HA-bs* (Fig. 2B), although we noticed that the rescued myofibrils become greater in diameter than those of controls (Fig. 2B), suggesting that higher levels of Bs might promote the formation of larger myofibrils. For sarcomere length, control sarcomeres grew from ∼1.5 µm at 48 h APF to ∼3.5 µm. The sarcomere lengths of *bs* KD samples was essentially the same as controls at both time points (Fig. 2C). These results indicate that *bs* is not required to set up the initial architecture of the IFM myofibrils nor for the initiation of myofibril assembly. Instead, *bs* is required for the normal growth of myofibrils during IFM differentiation.

### The muscle phenotypes of *bs* KD are isoform-specific

**Fig. 3.**
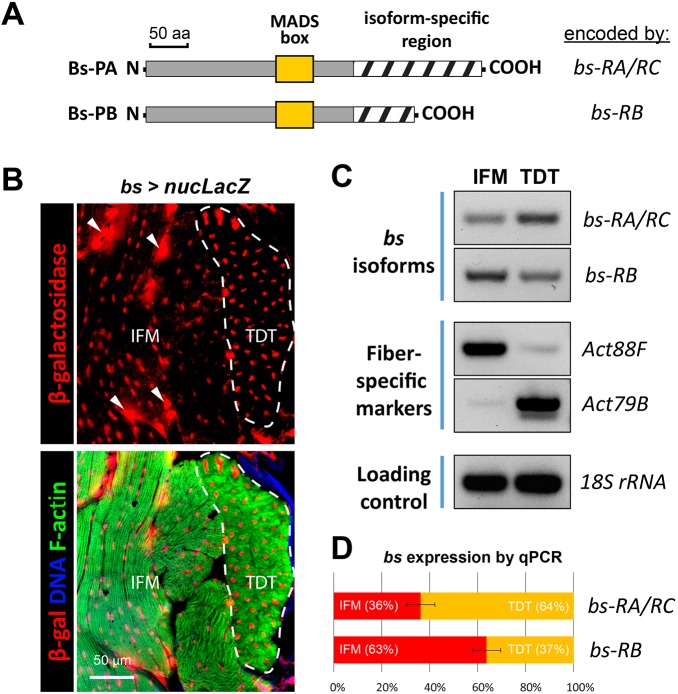
**Expression of *bs* isoforms in flight and jump muscles.** (A) Schematic representation of structural differences of the two annotated Bs protein isoforms. The MADS box is a DNA-binding domain. (B) Activity of the *bs* locus driving expression of nuclear *LacZ* reporter (*nucLacZ*) in the enhancer-trap line BDSC25753. Reporter-produced nuclear β-galactosidase was detected by immunofluorescence (red) in addition to F-actin (green) and nuclear (blue) counterstaining that was added to the lower panel. Jump muscle is outlined; arrowheads indicate tracheal cell nuclei outside muscles. (C) Expression of *bs* isoforms in isolated IFM and TDT, as detected by RT-PCR. Fiber-specific markers (*Act88F* and *Act79B*) and ribosomal RNA (18S rRNA) validate sample purity and equal loading. (D) Expression *of b*s isoforms between IFM and TDT muscles, as determined by qPCR. Data are mean±s.d. (three biological repeats).

To further investigate the IFM specificity of *bs* KD and to define the contributions of the different Bs isoforms to muscle differentiation, we studied the isoform-specific expression and roles of Bs. According to its gene model, the *bs* locus produces three annotated transcripts (Fig. 1B). Transcripts *bs-RA* and *bs-RC* have identical coding capacity, resulting in the protein isoform Bs-PA; transcript *bs-RB* is alternatively spliced and encodes protein isoform Bs-PB (Fig. 3A). Both protein isoforms share the identical N terminus and the DNA-binding MADS domain. However, their C-terminal regions involved in transactivation activities (Liu et al., 1993) have different sequences and lengths (Fig. 3A). When comparing the unique C-terminal sequences with the protein database, we did not identify regions of predicted function.

To further confirm *bs* expression in adult muscles, we employed a genetic approach: a previously described *bs* enhancer-Gal4, comprising 4.4 kb of genomic DNA (Guillemin et al., 2001), was used to express nuclear β-galactosidase. We detected strong β-galactosidase accumulation in the nuclei of tracheal cells (arrowheads in Fig. 3B), in agreement with previous data (Guillemin et al., 1996). β-Galactosidase was also detected in adult muscles. Importantly, flight and jump muscles were equally positive for β-galactosidase (Fig. 3B). This suggested that *bs* expression in adult flies is not limited to a specific muscle. Data from additional experiments in this study supported this conclusion, as *bs* expression was detected by RT-PCR in isolated flight and jump muscles (see Figs 1B, 3C and 7C).

To examine whether expression of Bs isoforms is fiber specific, we prepared RNA samples from dissected flight and jump muscles, for which purity was confirmed by assaying the expression of fiber-specific genes using end-point RT-PCR (Bryantsev et al., 2012b). We detected transcripts for both Bs-PA and Bs-PB isoforms in each muscle sample (Fig. 3C); using qPCR, transcripts encoding Bs-PA were more abundant in TDTs than in IFMs, whereas Bs-PB-encoding transcripts were slightly enriched in IFMs (Fig. 3D). Collectively, these findings suggest that both SRF isoforms can have muscle-specific roles.

The Bs-PB isoform was not targeted by RNAi in our original screen or by the mutant analysis. To determine whether this isoform has a muscle-specific function, we investigated its role via a separate KD. We created an RNAi construct (termed AD1) targeting the unique sequence of the *bs-RB* transcript (Fig. 1B). The efficacy of the AD1 RNAi construct was tested using a viability assay, as Bs is required for viability through an essential role in tracheal development (Affolter et al., 1994). Importantly, lowering Bs-PB levels in developing embryos by crossing AD1 with the trachea-specific driver *btl-Gal4* (Shiga et al., 1996) resulted in lethality. This result confirmed the function of the construct (Fig. S1A). Knocking down Bs-PA (via the KK108659 RNAi construct) with the *btl-Gal4* driver also resulted in lethality (Fig. S1A).

When AD1 was used to knock down Bs-PB in developing adult muscles (via the *1151-Gal4* driver), the flies eclosed normally and had normal performance in muscle functional assays (Fig. S1B), in contrast to Bs-PA, which was crucial in the functioning of the flight muscle (Fig. S1B) and partially required in the functioning of the jump muscle (Fig. S1B). We conclude that although both Bs isoforms contribute to critical aspects of organismal organization, only the longer Bs-PA isoform has a detectable role in adult *Drosophila* muscles. Although it is possible that overexpression of the PB isoform could rescue the mutant phenotypes we observe from KD or mutation of the PA isoform, we have found no evidence to suggest a role for the PB isoform in muscle formation.

### Genome-wide identification of genes whose expression is affected by *bs* KD

**Fig. 4.**
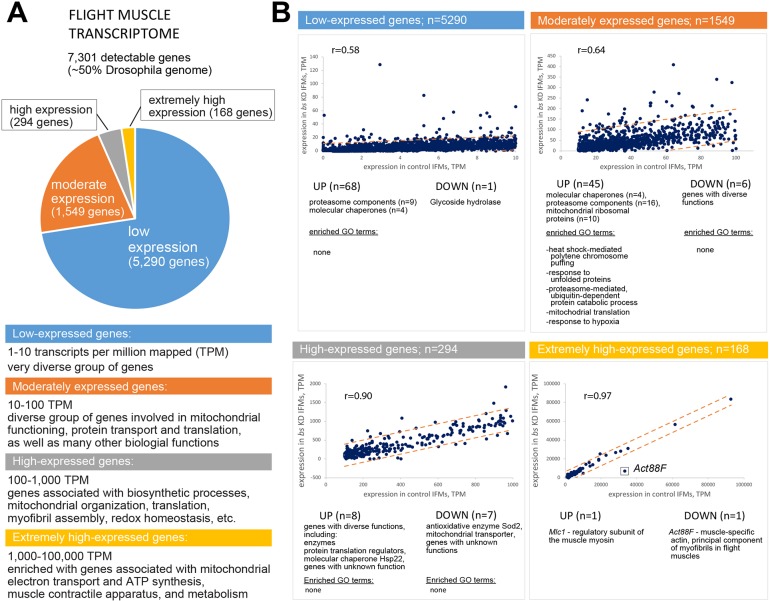
**Changes in the flight muscle transcriptome in response to *bs* knockdown.** (A) Distribution and characterization of transcriptionally active IFM genes grouped into expression tiers. (B) Identification and brief characterization of genes with significantly altered expression levels. Dotted lines delineate the confidence interval (±3 standard deviations from the regression line). r, Pearson's correlation coefficient. The box indicates the position of *Act88F* in the expression coordinates.

To identify the putative gene targets of Bs within the flight muscle, we prepared total RNA samples extracted from dissected flight muscles of 3-day-old flies and submitted for RNAseq analysis. Illumina sequencing produced reads that reliably mapped to a total of 7301 genes, approximately half of all annotated *Drosophila* genes (see Table S2). We assigned IFM-expressed genes into four tiers, based on expression levels in the control IFM (Fig. 4A). The vast majority of the genes (*n*=5290) were assigned to the low-expressing tier, where the transcription rate did not exceed 10 transcripts per million of mapped transcripts (TPM). The moderately expressing tier (*n*=1549) contained genes with expression rates in the range of 10-100 TPM. The other two tiers with high (100-1000 TPM) and extremely high (1000-100,000 TPM) expression rates had low numbers of gene members (*n*=294 and *n*=168, respectively). From Gene Ontology (GO) analysis, it is evident that IFM-expressed genes are involved in a wide spectrum of biological functions; however, the most transcriptionally active genes are associated with mitochondrial functioning, metabolic processes and myofibril assembly (Tables S3 and S4).

We plotted transcriptional activity of each gene using TPM coordinates supplied by control and *bs*-KD IFM samples (Fig. 4B). Genes whose expression deviated by greater that three standard deviations from the regression line were considered to show significantly altered expression. According to this statistical cut-off, 137 genes (less than 2% of all mapped genes) showed significant expression alterations upon *bs* KD (Table S3), and only 15 genes were identified as downregulated. As *bs* KD drastically reduced myofibril diameter in IFMs, we were most interested in finding genes that underwent downregulation in the extremely high-expressing tier, which, according to our GO term enrichment data, contains genes involved in myofibril assembly (Table S4). Indeed, the only downregulated gene in that group was identified as *Act88F*, the IFM-specific muscle actin (Fyrberg et al., 1983). In fact, *Act88F* was the most significantly downregulated outlier in the entire IFM transcriptome (Fig. 4B). Other downregulated genes were those with diverse functions and could not be consolidated based on common parameters. In contrast, among the upregulated genes we identified overrepresented groups involved in unfolded protein response, including chaperones, proteasomal components and protein translation machinery components. Many of these genes have recently been reported to become upregulated in the IFM of *Act88F*-null mutants (Madan et al., 2017; Table S3). It is likely that these genes respond in a stereotypic way to handle the surplus of myofibrillar proteins caused by muscle actin shortage (see Fig. 2A, arrowheads); therefore, they may not be direct Bs targets. Notably, our list does not have a substantial presence of myofibrillar components, besides a weak surge in the expression of myosin light chain *Mlc2* (which was not confirmed by an alternative statistical analysis, see Tables S2 and S3), and in the expression of low-expressing muscle actin *Act57B* and troponin *TpnC41C*, both of which are not IFM specific. Further transcriptome studies employing multiple biological repeats should be more potent in determining which genes consistently respond to *bs* KD; nevertheless, our analysis clearly identifies *Act88F* as a major target of Bs in the flight muscle.

### The Bs-PA isoform selectively affects expression of the fiber-specific actin *Act88F*

**Fig. 5.**
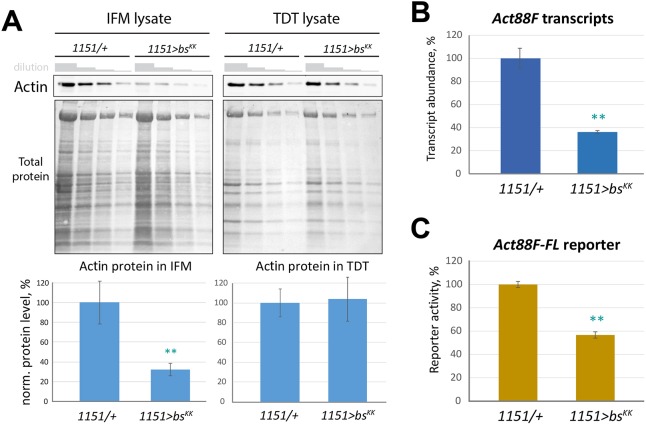
***bs* knockdown affects *Act88F* expression.** (A) Semi-quantitative western blots prepared with serially diluted protein lysates from isolated IFMs and TDTs, dissected from control (*1151/+*) and *bs* KD (*1151>bs^KK^*) flies. Gray step-slopes show the dilution rate (factor of 2). The membranes were stained using anti-actin antibody (upper panels) as well as general protein dye (lower panels). Intensities of the upper panel bands were normalized to intensities of eight most prominent bands from the lower panels and are presented on the graphs below. (B) Abundance of *Act88F* transcripts in isolated IFMs, as determined by qPCR. (C) Activity of the full-length, cloned *Act88F* enhancer driving *LacZ* expression. *LacZ* expression levels were quantitatively determined in whole-fly lysates by liquid β-galactosidase assay. All data are an average of three or four independent measurements or assays±s.d. Student's *t*-test was used to compare values between control and *bs* KD samples. **P<0.01.

To validate the RNAseq finding, we analyzed actin protein levels in *bs* KD adult muscles. IFM and TDT muscles were dissected from control and *bs* KD animals, and analyzed for actin accumulation using western blotting. For *bs* KD animals, actin levels were reduced to ∼30% of controls, whereas actin levels were unaffected in the TDT (Fig. 5A). As the IFMs and TDTs accumulate different actin isoforms (Act88F and Act79B, respectively; Ball et al., 1987; Courchesne-Smith and Tobin, 1989), these results suggested that *bs* KD affected the expression of *Act88F*. To test this, we carried out quantitative RT-PCR for *Act88F* transcripts relative to controls, and found that, in *bs-RA* KD flight muscles, *Act88F* transcripts significantly diminished to about 40% of the level seen in controls (Fig. 5B). Therefore, these findings are in full agreement with our results from the transcriptome analysis.

To determine whether the observed reduction of *Act88F* transcript levels was due to changes in transcriptional control of this gene, we employed a genetic reporter assay. We used our previously described *Act88F-FL* reporter, in which a 1 kb genomic element cloned from the region immediately upstream of the *Act88F* gene drives strong expression of β-galactosidase in the IFMs of transgenic animals (Bryantsev et al., 2012a). In Bs-PA KD, the activity of *Act88F-FL-LacZ* was substantially reduced, closely resembling the change in the expression of endogenous *Act88F* transcripts (Fig. 5B,C). Based on these transcriptional profiling and reporter assay results, we conclude that Bs-PA has a role in *Drosophila* flight muscles by controlling expression of the IFM-specific actin gene *Act88F*.

### Bs binds specifically to the *Act88F* promoter

**Fig. 6.**
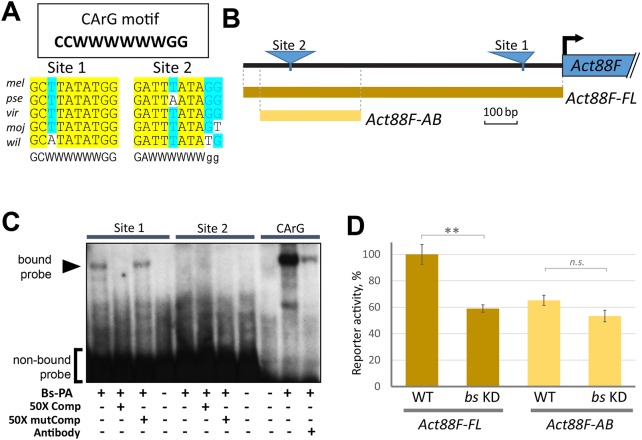
**Bs directly binds to the *Act88F* enhancer.** (A) The two most conserved putative Bs-binding sequences within the *Act88F* enhancer. Sites 1 and 2 share sequence similarities with the canonical CArG motif (framed, W=A/T). Yellow highlight indicates nucleotides with absolute conservation across eight distantly related *Drosophila* species, including: *D. melanogaster* (*mel*), *D. pseudoobscura* (*pse*), *D. virilis* (*vir*), *D. mojavensis* (*moj*) and *D. willistoni* (*wil*). Blue highlight indicates positions with significant conservation that contained mismatches in not more than two species. (B) Schematics of the full-length cloned *Act88F* enhancer (*Act88F*-*FL*) and its truncated version (*Act88F*-*AB*), with locations of putative Bs-binding sites. (C) Electrophoretic mobility shift assay (EMSA). Nuclear extracts from S2 cells transfected with a Bs-PA expression plasmid were examined for protein/DNA complex formation (marked with arrowhead), using radiolabeled probes representing site 1, site 2 or classical CarG sequences. Essential components of binding reactions are indicated, including Bs protein (Myc-tagged Bs-PA isoform), a 50-fold excess of non-labeled probes (intact or mutated) and anti-Myc antibody, which were used to validate binding specificity. DNA/protein complex formation is seen only with site 1 and CarG probes, and it is specific to Bs protein presence. (D) *In vivo* activities of two *Act88F-LacZ* reporters, bearing the enhancers depicted in B. Data were obtained in whole-fly lysates and represent averaged β-galactosidase activity from three independent assays (±s.d.). *t*-test results: **P<0.01; n.s., *P*>0.05.

The fact that *Act88F-FL* was sensitive to *bs* KD in muscles suggested that the 1 kb promoter/enhancer present in this reporter contains Bs-dependent regulatory elements. Bs shares 95% homology with the MADS DNA-binding domain of mammalian SRF (Pellegrini et al., 1995), a sequence-specific transcription factor that recognizes and binds to the DNA motif called the CArG box (consensus 5′-CCWWWWWWGG) (Minty and Kedes, 1986; Fig. 6A) as well as its sequence variants. CArG boxes bind SRF homologs across distant phyla, including crustaceans (Casero and Sastre, 2001; Shore and Sharrocks, 1995). According to a bioinformatic analysis, the sequence of the *Act88F-FL* promoter/enhancer does not contain the classical CArG box. However, we identified 30 instances of the CArG core sequence, which is represented by a short stretch of six A and/or T nucleotides (designated as W in the CArG motif, Fig. 6A). To narrow down those sites that may bind the Bs protein and therefore be functional, we determined the evolutionary conservation around the identified CArG core sequences. By aligning the *Act88F* 1 kb sequence with homologous genomic regions from multiple *Drosophila* species (Drosophila 12 Genomes et al., 2007), we confirmed substantial conservation for only two sites (sites 1 and 2, Fig. 6A). Site 1 was located close to the TATA-box and transcription start site, while site 2 was located more distally (Fig. 6B).

To determine whether Bs could bind specifically to these sites, we carried out an electrophoretic mobility shift assay (EMSA). According to this assay, Bs bound to site 1 but not to site 2 (Fig. 6C). Interestingly, EMSA also revealed a lower binding affinity of Bs to site 1 in comparison with the classical CArG motif. This may highlight the functional consequence of a single-nucleotide substitution within site 1, in which a minor mutation is evolutionarily fixed in the promoters/enhancers of flight muscle-specific actin genes across all tested *Drosophila* species (Fig. 6C).

To define *in vivo* the requirement in gene expression of the sequences including site 1, we generated a shorter enhancer, termed *Act88F-AB*, that lacked the promoter-proximal sequences containing site 1 (Fig. 6B). Transgenic flies bearing the *Act88F-AB* reporter, inserted at the same genomic position as in *Act88F-FL* transgenic flies, were analyzed in a quantitative β-galactosidase assay. In a wild-type genetic background, the transcriptional activity of *Act88F-AB* was approximately half of that of *Act88F-FL* (Fig. 6D). Notably upon *bs* KD, *Act88F-AB* activity did not significantly change, whereas *Act88F-FL* activity became reduced to the levels characteristic of *Act88F-AB* (Fig. 6D). These results indicate that Bs controls *Act88F* gene expression *in vivo* via the region containing site 1.

Altogether, our data demonstrate that the enhancer/promoter of the IFM-specific actin gene *Act88F* contains at least one functional binding site for Bs. Our results also indicate that the transcriptional activity of the *Act88F* gene depends partially, albeit substantially, on Bs.

### Integration of Bs-dependent regulation into the global control of *Act88F*

The fact that the expression of Bs in muscles occurs in a broader domain than the expression of its target, the flight muscle-specific *Act88F*, implies the existence of an additional level of regulation for the *Act88F* promoter/enhancer. In a previous study, we found that a heterodimeric complex formed by the homedomain transcription factors Homothorax (Hth) and Extradenticle (Exd) directly binds and activates the *Act88F* enhancer (Bryantsev et al., 2012b). Thus, we hypothesized that the Exd/Hth complex might cooperate with Bs in the regulation of *Act88F*. To test this hypothesis, we determined critical developmental time frames required for either Hth or Bs to influence *Act88F* expression.

**Fig. 7.**
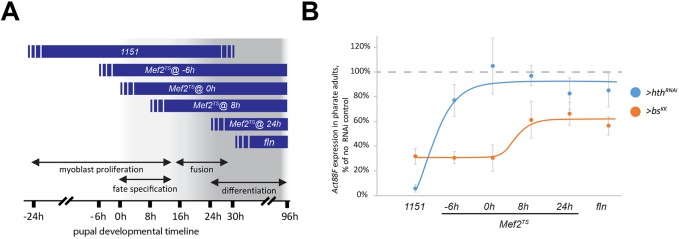
**Expression of *Act88F* during adult myogenesis depends on** ***exd*, *hth*** **and *bs*, but at temporally distinct stages.** (A) Schematics of pupal developmental timeline, indicating important phases of IFM development (double arrows) and activity windows for various genetic drivers (blue ribbons) used for knocking down *hth* and *bs* genes. The Mef2^TS^ driver is temperature sensitive, which allows deliberate activation at different times, as indicated. Exact timing of each driver is approximate, but the chronological order of different drivers is maintained. (B) Effects of *hth* (blue) and *bs* (orange) knockdowns, induced at various timepoints of development, on the expression of *Act88F.* Quantification of *Act88F* transcripts was performed by qPCR in pharate adults and is expressed as percentage of similarly treated genetically matched controls. Each data point is an average from three or four biological repeats±s.d. Solid lines show the approximated trend. *Act88F* expression depends on *hth* only at very early stages of IFM development, whereas *bs* retains control over *Act88F* for the entire period.

In a first experiment, we used Gal4 drivers that become active at different stages of muscle development to knock down expression of *hth* or *bs-RA/RC* (Fig. 7A). We used the *1151-Gal4* driver, which is first active in myoblasts prior to muscle differentiation (Roy and VijayRaghavan, 1998), and the *fln-Gal4* driver, that is active only after the onset of muscle differentiation (Bryantsev et al., 2012b). *Act88F* expression levels for all treatments were analyzed in pharate adults. With the *1151-Gal4* driver, *Act88F* transcript levels in the flight muscles were strongly reduced when we knocked down either *hth* or *bs* and, characteristically, *hth* KD had a greater impact on *Act88F* expression (Fig. 7B). By contrast, using the *fln-Gal4* driver, KD of *hth* had only a minimal effect upon *Act88F* transcript levels, yet KD of *bs* resulted in a strong reduction in *Act88F* transcript levels (Fig. 7B). These results indicate that *hth* expression must be knocked down early during muscle formation to impact IFM fate and *Act88F* expression, whereas *bs* KD starting at either early or late stages can significantly impact *Act88F* expression.

In a second experiment, we used a temperature-sensitive expression system to knock down the expression of *hth* or *bs* at distinct times of adult muscle development. We generated stocks that carried: the *Mef2-Gal4* driver, which is active in myoblasts and muscles throughout development (Ranganayakulu et al., 1998); the temperature-sensitive *Gal80^ts^* gene, which encodes a repressor of Gal4 function that can be inactivated by increasing the incubation temperature (McGuire et al., 2003); and a UAS-RNAi targeting – either *hth* or *bs*. Specifically, the incubation temperature was increased to initiate the KDs, starting at different developmental times, and the KD was sustained for the remainder of pupal development. Pharate adults were then analyzed for *Act88F* transcript levels (see Materials and Methods, Fig. 7A).

We found that if *hth* KD was initiated before the beginning of adult myogenesis (-6 h apf) and sustained for the remainder of the pupal stage, *Act88F* expression was significantly reduced (Fig. 7B). This was in agreement with the results with the *1151* and *fln* Gal4 drivers, and with our published studies demonstrating that Hth/Exd complex is expressed in founder cells, before the initiation of myoblast fusion (Bryantsev et al., 2012b). When we delayed the onset of *hth* KD for a few hours, the negative effect of *hth* KD on *Act88F* expression was entirely abrogated (Fig. 7B). This was not due to high stability of Hth protein, because KD of *hth* at 0 hr resulted in a clear absence of Hth protein at 48h APF (Fig. S2). These findings indicated that the most critical time at which Hth is required for flight muscle development is during early muscle specification.

For *bs*, induction of KD at all time points compromised *Act88F* expression (Fig. 7B). These data indicate that the requirement for *bs* in *Act88F* expression is later than that for *hth*, suggesting that *bs* functions temporally downstream of the flight muscle-specification factors.

**Fig. 8.**
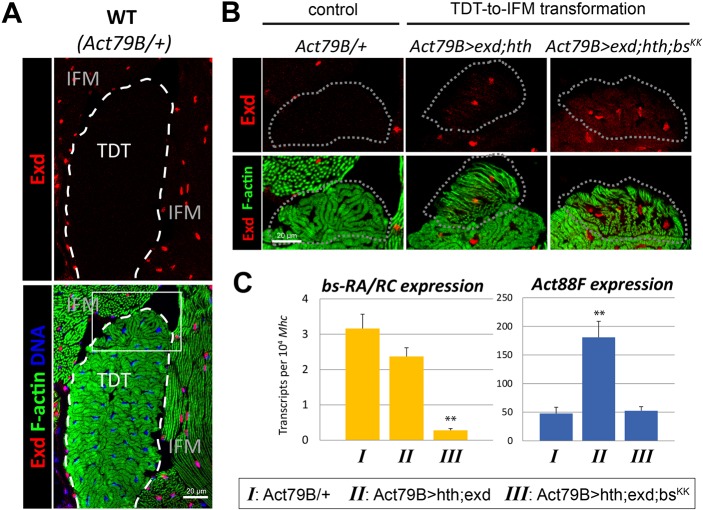
**Flight muscle identity factors *hth* and *exd* rely on *bs* for sustained *Act88F* expression.** (A) Exd expression in thoracic muscles. Cryosections of pharate adult thoraces were immunolabeled for Exd protein (red) and counterstained for actin myofibrils (green) and DNA (blue). The boxed area is shown at higher magnification in B. Exd protein (representing Exd/Hth complex) is not detectable in the jump muscle (TDT, outlined), but is well-expressed in nuclei of flight muscles (IFM). (B) Experimental identity conversion of jump muscle fibers. Wild type (WT), normal condition of small jump muscle cells (outlined). Expression of *UAS*-*exd* and *UAS*-*hth* transgenes (*Act79B>exd;hth*) was achieved using a jump muscle-specific *Act79B-Gal4* driver and confirmed by nuclear Exd accumulation (red). Exd-expressing fibers demonstrate altered morphology of myofibrils (green) that indicates fiber type conversion. When *bs* knockdown was introduced (*Act79B>exd;hth;bs^KK^*), it did not affect Exd nuclear localization or fiber type transformation. (C) Quantification of *bs*-*RA* and *Act88F* expression upon experimental fiber fate conversion. Transcript abundance was determined by qPCR in samples obtained by microsampling from the regions outlined by the dotted line in B. *Myosin heavy-chain* (*Mhc*) was used for transcript normalization. Each bar represents the average of three measurements±s.d. **P<0.01, using Student's *t*-test.

The above results imply that *Act88F* expression will not be activated by Bs if the Exd/Hth complex is not present. Indeed the jump muscle naturally lacks the Exd/Hth complex but retains Bs (Fig. 8A and Fig. 3A, respectively), and *Act88F* is not actively expressed in the mature jump muscles (Fig. 3C), although *Act88F* is transiently expressed in the small cells of the TDT during pupal development (Dohn and Cripps, 2018). To investigate this experimentally, we ectopically expressed the Exd/Hth complex in developing jump muscles, to study whether it is sufficient to activate *Act88F*. Importantly, for this experiment we used *Act79B-Gal4*, which is a late jump muscle-specific driver that is activated after myoblast fusion (Bryantsev et al., 2012a), and therefore the transformation of the jump muscle into flight muscle is not complete. Muscle tissue samples were obtained from cryosections by using a microscope-controlled microdissecting technique, to ensure the accuracy of the analysis (Fig. 8B, outlined). Following RNA extraction, gene expression was analyzed using quantitative RT-PCR. Using this approach, we confirmed our earlier observation that *bs* transcripts encoding the Bs-A isoform are expressed in normal jump muscles at a detectable level, which was not strongly affected upon the introduction of the Exd/Hth complex (Fig. 8C, left panel).

Ectopic expression of *exd* plus *hth* in jump muscles increased basal *Act88F* levels by approximately fourfold (Fig. 8C, right panel), consistent with the ability of these factors to bind to and activate the *Act88F* promoter/enhancer. By contrast, when we induced *bs* KD in the jump muscles expressing *exd* plus *hth*, the levels of *Act88F* transcripts returned to basal levels (Fig. 8C, right panel). These experiments demonstrate that Bs is required for Exd/Hth to activate *Act88F* expression in the jump muscles.

Overall, our data indicate that the Exd/Hth complex functions early to initiate expression from the *Act88F* promoter/enhancer, while Bs is necessary to sustain this expression. Importantly, Bs on its own is not sufficient to initiate high levels of *Act88F* expression *in vivo*.

## DISCUSSION

### A missing evolutionary link in muscle-specific gene regulation by SRF

Our study is the first report demonstrating that a non-vertebrate SRF is directly involved in transcriptional regulation of muscle actin gene expression. SRF is a MADS domain transcription factor and our studies demonstrate that, as in vertebrates, both SRF and another MADS domain transcription factor, MEF2, are crucial for normal adult muscle development (Bryantsev et al., 2012a). Members of the SRF family are thought to have developed as regulators of cytoplasmic actin genes. Given the importance of actin gene regulation for all eukaryotic organisms, SRF family members have been identified in practically all eukaryotes studied (Miano et al., 2007). Concomitant with the function of cytoplasmic actin, SRFs have been shown to affect cell migration and shape (e.g. see Schratt et al., 2002, 2001). Although direct genetic regulation of the *Drosophila* cytoplasmic actin genes *Act5C* and *Act42A* has not been demonstrated for *Drosophila* SRF, *Act5C* contains CArG boxes in its two alternate promoters (Bond-Matthews and Davidson, 1988). Moreover, *bs* is required for normal tracheal and wing-vein branching, processes that are likely to be actin-dependent (Affolter et al., 1994; Fristrom et al., 1994). In our RNAseq profiling, neither *Act42A* nor *Act5C* demonstrated significantly altered expression in response to *bs-RA/RC* KD in IFMs; however, we cannot rule out the possibility that these actin genes more readily respond to *bs-RB* isoform.

Another function attributed to SRF is the transcriptional regulation of muscle-specific actins. This function has been described exclusively for vertebrate organisms that have multiple muscle-specific actin genes (Miano et al., 2007). Until this study, a role for non-vertebrate SRFs in muscle has only been demonstrated in *C. elegans*, despite the existence of multiple actin genes in many invertebrates (e.g. see Adema et al., 2017). *Drosophila* appeared to be an outlier, given that embryos null for *bs* function did not show overt muscle phenotypes (Affolter et al., 1994). Our study fills in this knowledge gap by demonstrating that indeed Bs is involved in direct regulation of *Act88F* – one of the four muscle-specific actin genes in *Drosophila*. These findings indicate a deep evolutionary role for SRF in regulation of myofibrillar actin transcription. Nevertheless, unlike the situation in vertebrates (Miano et al., 2007), our transcriptome data do not indicate that Bs regulates the expression of numerous myofibrillar protein genes, at least in the flight muscles, which may instead be a later evolutionary innovation. Indeed, the relatively short list of Bs-dependent transcripts in the flight muscles seems surprising given the broad expression of this gene in muscles. It is possible that, as in *C. elegans*, *Drosophila* SRF functions redundantly with other factors. Alternatively, the time-point for RNA isolation in our experiments may not match the developmental time at which Bs exerts a strong influence over a large number of target genes. Therefore, additional Bs targets might be uncovered by transcriptome analysis at serial time points.

### A fiber-specific role for SRF in *Drosophila*

The requirement for *Drosophila* SRF in muscle shows a striking restriction to the fibrillar fiber type: other adult muscles appear to be unaffected by *bs* KD, and actin levels are unaffected in the jump muscles of *bs* KD animals. This result is somewhat reminiscent of the studies of Charvet et al. (2006), where a knockout of SRF in skeletal muscles of adult mice resulted in a failure of further muscle growth and a failure of muscle regeneration. Importantly, mutant muscles showed a reduction in fast/type IIB fibers, presumably resulting from a fast-to-slow transition in fiber identity. Although fiber-type switching cannot be directly equated to differences in fiber-type differentiation, our data, in combination with those of Charvet et al., imply that fiber-specific roles for SRF may exist in vertebrate skeletal muscles.

Although the predominant effects of *bs* KD appear in the flight muscles, we note that jumping ability is reduced in *bs* KD animals compared with controls (see Fig. S1). This is clearly not due to reduced actin accumulation in the KD TDTs (see Fig. 5), and may result from a requirement for Bs in the expression of genes, yet to be identified, that contribute to jump muscle function. Alternatively, defects in flight muscle structure may affect the flexibility or rigidity of the thorax, and this reduced stiffness could dampen the jumping power. Some support for this latter explanation comes from the reduced jumping ability of null mutants for *TpnC4*, an IFM-specific Troponin C gene (Chechenova et al., 2017).

### Exd and Hth may function as pioneer factors in muscle identity

We identified only a single CArG site in the *Act88F* enhancer/promoter. Moreover, this site deviates from the canonical CArG motif by a single nucleotide, which apparently lowers its affinity to Bs. It would be interesting to mutate this site in the context of the enhancer-*LacZ* to test whether it is essential for *Act88F* expression, and to determine whether other non-consensus sites might be present. CArG sites are generally present in pairs or small clusters (Blank et al., 1992; Miano, 2003), which could be a redundant feature or could serve to better recruit co-factors. Single CArG sites in mammals have also been reported (Miano, 2003).

**Fig. 9.**
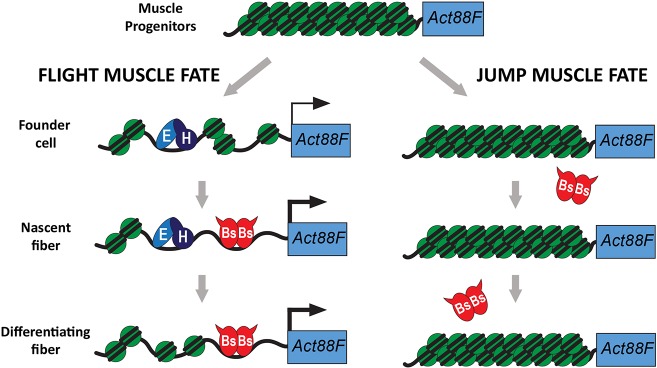
**Model for *Act88F* transcriptional initiation and maintenance.** In adult muscle progenitor cells, no expression of the *Act88F* gene (blue box) is evident, as indicated by the condensed chromatin state of its promoter/enhancer. In muscle precursors, upon specification as flight muscles, the pioneer factors Exd and Hth bind to the *Act88F* enhancer and loosen the chromatin to provide initial activation of *Act88F* transcription (thin line with arrow). In nascent flight muscles, Bs binds to the CarG box in the proximal promoter and boosts *Act88F* transcription (thick line with arrow). Later, in differentiating fibers, the Exd/Hth complex may no longer be present at the enhancer, but Bs maintains a high level of transcription from *Act88F*. In contrast, in developing jump muscles, Bs is unable to access the CarG box without Exd and Hth, so the *Act88F* gene remains inactive.

An explanation for the single CArG box in *Act88F* could arise from a role for Bs in sustaining, rather than initiating, *Act88F* expression. For Bs to contribute to *Act88F* transcription, the cells must also be expressing (or have expressed) the homeodomain transcription factors Exd and Hth. We propose that Exd and Hth have an early function in opening up the chromatin in the vicinity of *Act88F*, after which Bs binds to the *Act88F* promoter to sustain its transcription (Fig. 9). This model is supported by the following evidence: (1) Bs alone is not sufficient for *Act88F* transcription in the jump muscle (Fig. 3); (2) Exd and Hth are dispensable for *Act88F* expression following the onset of flight muscle differentiation, whereas Bs remains required for a longer time (Fig. 7); and (3) Exd- and Hth-dependent ectopic expression of *Act88F* in the jump muscles is dependent upon *bs* (Fig. 8). Exd and Hth may therefore function as pioneer factors, promoting the transcriptional programming of specific cell types (see Iwafuchi-Doi and Zaret, 2014; Zaret and Carroll, 2011). This hypothesis needs to be tested by analyzing *Act88F* chromatin organization in control and mutant muscles; nevertheless, the mammalian Exd and Hth orthologs Pbx1 and Meis1 have been shown to function as pioneer factors in other contexts (Berkes et al., 2004; Heidt et al., 2007; Magnani et al., 2011). Exd and Hth also function to upregulate *salm* expression (Bryantsev et al., 2012b), and *salm* in turn is both necessary and sufficient to promote flight muscle fate (Schönbauer et al., 2011). An alternative to our hypothesis is that Salm may function as the pioneer factor.

One common feature of actin regulatory elements is the presence of cis-regulatory regions in proximity to the CArG sites. In vertebrates, proximal sites include E-boxes that bind bHLH transcription factors (Blank et al., 1992). In *Act88F* regulation, the Exd and Hth, and Bs binding sites are about 700 bp apart. This suggests that Exd and Hth are not acting directly with Bs, but rather cooperate with Bs by making site 1 available for Bs binding, presumably by loosening the chromatin around the CArG box.

### Cooperation of SRF with co-factors

In the context of muscle, SRF partners include Myocardin and other members of the MRTF family (Miano et al., 2007), and *Drosophila* has an MRTF ortholog (Han et al., 2004). Moreover, MRTFs may determine the selectivity of transcriptional activation by SRF complexes (Small, 2012). Knockdown of *Drosophila Mrtf* in myoblasts completely abolishes formation of all adult muscles (A.B. and R.M.C., unpublished), which supports its involvement in the early steps of myogenesis. This makes it less likely to be involved in fiber-specific differentiation, although MRTF may have distinct early and late functions in adult myogenesis.

Selective interaction with additional transcriptional partners may explain the different roles that we observed for Bs-A and Bs-B isoforms toward tracheal and muscle development. The DNA-binding abilities of the two dSRF isoforms are presumably the same, but variable C-terminal domains may provide an assembly point for distinct co-factors. It is also possible that the two isoforms compete with each other or have overlapping redundant roles that are yet to be uncovered.

## MATERIALS AND METHODS

### Fly stocks and husbandry

The following genetic fly lines were purchased from the Bloomington *Drosophila* Stock Center (BDSC) or the Vienna *Drosophila* Stock Center (VDRC): *Df(2R)exel6082/CyO* (BDSC 7561), *Df(2R)BSC603/SM6a* (BDSC 25436), *UAS-bs^KK108659^* (VDRC 108659), KK-library isogenic control (VDRC 60100), *UAS-exd^KK107437^* (VDRC 100687), *UAS-hth^HMS01112^* (BDSC 34637), *CyO/btl-Gal4* (BDSC 8807) and *bs*-*Gal4* (BDSC 25753). VDRC RNAi lines have been described previously (Dietzl et al., 2007), and the BDSC RNAi line is from Ni et al. (2011). The *bs* rescue flies *UAS-bs-HA* (F001824) were from FlyORF (flyorf.ch). The TDT-specific driver line *Act79B*>*Gal4* has been described previously (Bryantsev et al., 2012a).

To create the temperature-sensitive driver line Mef2^ts^, standard crossing techniques were used to make a stock harboring *Mef2-Gal4* (III) (a gift from A. Johnson, Washington University in St. Louis, MO, USA) and *tub-Gal80^TS^* (BDSC 7108). The *fln-Gal4* line is a modification of the *fln*-*nLacZ* transgenic line described by us previously (Bryantsev et al., 2012b), with the exception that the *fln* enhancer drives Gal4 expression. The pattern of expression of the *fln-Gal4* was verified to be exclusively IFM specific, as reported before for *fln-LacZ*.

The myoblast-specific driver line *1151-Gal4* was a gift from L. S. Shashidara (Indian Institute of Science Education and Research, Pune, India). *Act88F-FL* and *Act88F-AB* reporter lines were created using the identical protocol: the *Act88F* enhancer sequences were amplified (see primer sequences below) and cloned into pDONR-nLacZ-attB, a Gateway-compatible derivate of a *LacZ* reporter described elsewhere (Bischof et al., 2007). Importantly, the FL and AB reporters were integrated at the same attP genomic landing site (located in the cytogenetic locus 86Fb) of the parental line (BDSC 24749, see Bischof et al., 2007). Construct injections for transgenesis were performed by Rainbow Transgenic Flies.

Fly stocks were maintained on standard fly food (Jazz mix, Fisher) at 25°C. Fly crosses were set and maintained at 29°C, unless otherwise specified.

For the temperature-sensitive KD study, the crosses with Mef2^ts^ flies were reared at 18°C. Depending on the desirable time point of RNAi activation, late wandering larva, white pupae or staged (6 or 24 h) pupae were transferred to 29°C where the rest of their development took place.

### Making the *bs-RB* RNAi line

A 575 bp region, unique to the *bs-RB* transcript, was amplified (Table 1) and twice inserted into the assisting cloning vector pGEM-WIZ (Bao and Cagan, 2006) to obtain a tandem repeat with head-to-head orientation. Cloning details have been described previously (Bryantsev et al., 2012a). After construct orientation verification, the region containing the inverted copies, separated by an intronic spacer, was subcloned into pUASt (Brand and Perrimon, 1993) vector and used for transposon-mediated transgenesis, following standard techniques (Rubin and Spradling, 1982).

**Table 1.**
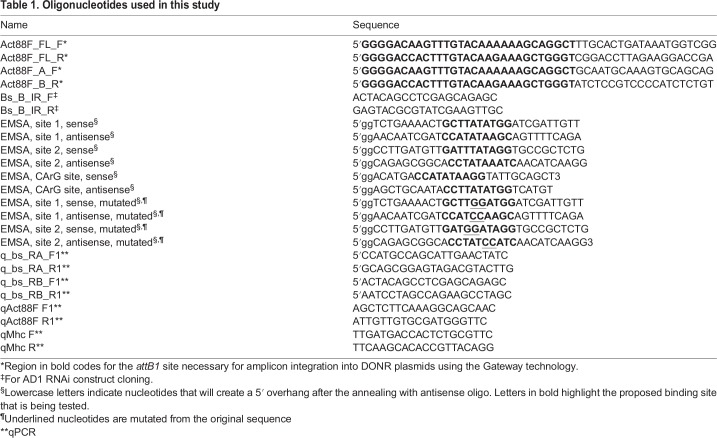
**Oligonucleotides used in this study**

### RNAi screening

The crosses between the driver line females (line *1151-Gal4*) and males of RNAi-inducing lines were set and kept at 29°C. For morphologic analysis, young adults aged 1-2 days post eclosion were collected and used for cryosectioning.

### Functional tests

Flight tests were carried out in a flight chamber as described previously (Chechenova et al., 2017). For flight index calculations, each tested fly received a score of either 3, 2, 1 or 0 depending on whether it flew upward, horizontally, downward in the chamber or could not fly, respectively. Jump tests were performed essentially as described previously (Chechenova et al., 2017). In each test, 50-70 young adult flies were analyzed 2-3 days post eclosion.

### Cryosectioning and fluorescence microscopy

General procedures were followed as described previously (Morriss et al., 2012). Young flies were embedded in Tissue-Tek medium and immediately flash-frozen in liquid nitrogen. Frozen blocks were sectioned using a Triangle Biomedical Sciences cryostat. Sections (10 μm) were air-dried on slides and fixed with 3.7% formaldehyde for 10 min before processed for immunostaining. Muscles were revealed with Alexa-488 conjugated phalloidin (Molecular Probes); nuclei were counterstained with DAPI (Sigma). Confocal images were obtained using a LSM710 microscope (Zeiss). The following primary antibody were used: mouse monoclonal antibodies against alpha-actinin (Developmental Studies Hybridoma Bank, 2G3-3D7, 1:50) and β-galactosidase (Promega, Z378A, 0000167492, 1:1000), and rabbit polyclonal antibody against Hth (Santa Cruz, sc-26187, 1:100). Secondary antibodies were Cy3-labeled goat anti-mouse and anti-rabbit (both from Jackson ImmunoResearch, 115-167-003 and 111-167-003, respectively; 1:400), and Alexa 488-labeled goat anti-mouse antibody (Invitrogen, A32723, TA252657, 1:400).

### Morphometric analysis

Confocal images of thoraces from cryosections, prepared and stained as described above, were obtained with a 63× oil-immersion objective and analyzed with the Zen software (Carl Zeiss). For each condition, we measured between 150 and 250 myofibrils from four to seven animals (Table S1). We note that our measurements, by absolute values, differ slightly from previously published data (e.g. see Spletter et al., 2018). Such discrepancy may be attributed to the differences in the fixation protocol, as other research groups opt for prolonged formaldehyde fixation prior to muscle sectioning (pre-fixation). In a separate test, when we used a pre-fixation protocol (3.7% formaldehyde fixation for 24 h at 4°C, as in Spletter et al., 2018), we were able to fully recapitulate previously published morphometric results (Fig. S3). The data presented in this paper for analysis of *bs* KD and control muscles were generated using the methods described in the previous section, including post-fixation.

### RNA-sequencing and expression analysis

For RNAseq analysis, the collected young adults were staged for 3 days at 29°C to minimize gene expression fluctuations that are typical of recently eclosed flies. For each sample, flight muscles were dissected from females in 1 M sucrose and immediately dissolved in the solubilizing buffer provided by the RNeasy RNA purification kit (Qiagen). Total RNA was extracted following the kit manufacturer's protocol, including a 15 min on-column DNAse I digestion. Purified total RNA (100-200 ng) was sent for sequencing to the Sulzberger Genome Center at Columbia University. RNA quality control and library preparation was performed at the same center. Approximately 30 million of single-end, 100 bp reads were produced with an Illumina 4000 instrument. Post-run transcription analysis was performed on the free public bioinformatics platform Galaxy (usegalaxy.org). Sequenced reads in the FASTA format were run by the mapping/quantifying algorithm Salmon (Patro et al., 2017), which used all annotated transcripts and gene coordinates from the 6.21 release of the *D. melanogaster* genome. Salmon produced consolidated transcript counts for each *Drosophila* gene in the ‘transcripts per million’ (TPM) metric. Preliminary analysis of gene expression differences between control and *bs* KD samples was performed using the DEseq2 method (Love et al., 2014;Table S2). To better visualize the expression variances and provide alternative, less stringent analysis, we split the expressing genes into four tiers, based on the magnitude of their transcriptional output, as explained in the Results, and applied the regression analysis for each group. To do that, a linear regression was calculated for genes, plotted in control versus *bs* KD TPM coordinates, and the minimal R distance was computed between the actual and the projected positions for each gene. The confidence interval for R distances was set to three standard deviations from the regression line.

### Real-time quantitative PCR

Muscles dissected as described for the RNA-seq procedure, or entire pharate adults (for Fig. 7), were used for total RNA extraction using the RNeasy RNA purification kit (Qiagen), followed by cDNA synthesis with SuperScript II kit (Invitrogen). cDNA mixes were diluted five times and used for real-time amplification with Power Sybr Green mix (Applied Biosystems) and the Prism 7000 instrument (Applied Biosystems). We used the absolute quantification method, where sample reactions were run alongside a series of purified and titrated amplicons of known concentrations; post-run analyses were carried out using the SDS software (Applied Biosystems). The abundance of *bs* and *Act88F* transcripts was normalized by the amount of *Mhc* transcripts in each sample (Table 1).

### Microsampling technique

This was carried out essentially as described previously (Bryantsev et al., 2019).

### Western blotting

For each genetic condition, eight freshly eclosed flies were used for IFM and TDT muscle isolation. Dissected muscles were homogenized in 30 μl of 2× Laemmli sample buffer and used to prepare three twofold serial dilutions. After 3 min boiling, the diluted samples were run on 4-20% gradient acrylamide gel and transferred onto nitrocellulose membrane. Membranes were stained with Ponceau S and photographed. After brief destaining and blocking with 2% non-fat milk, the membranes were labeled with primary rabbit anti-actin antibody (Cell Signaling, 4968S, 02/2018, 1:1000) and secondary goat anti-rabbit, HRP-conjugated antibody (Jackson ImmunoResearch, 111-035-144, 1:1500). Chemiluminescence imaging of membranes was performed using ECL chemistry (Pierce) with a ChemiDoc imager (Bio-Rad). Image Lab software (Bio-Rad) was then used to run band intensity quantification.

### β-Galactosidase activity assay

Young adult flies were frozen at 3 days post eclosion and kept at −20°C. Each fly was ground to homogeneity in 100 µl of PBS buffer containing 0.1% Triton X-100 and debris was pelleted at 14,000 ***g*** for 1 min. Clarified lysates were loaded onto a 96-well plate (80 µl/well) and mixed with β-galactosidase reagent (ThermoFisher Scientific, 20 µl/well) and immediately placed at 37°C in a multi-well plate reader (ThermoFisher Scientific Multiskan, FC #51119100). Absorbance of developing reactions was measured at 405 nm every 2 min for an overall 22 min period. Absorbance rates for individual reactions were determined based on plotted reaction kinetics. Three flies were used per line in each assay run; final data were the average of three separate assay runs.

### Electrophoretic mobility shift assay

Nuclear extracts were prepared from S2 *Drosophila* cell cultures (Invitrogen; not recently validated nor tested for contamination) transfected with either empty vector (plasmid pPAc-Pl), or a vector containing the coding sequence of *bs-RA* fused to a Myc tag. Cells were plated at 5×10^5^ cells/well in a 24-well plate and transfected with TransIT transfection reagent in serum-containing medium (Mirus Bio). Immunofluorescence analysis with anti-Myc antibody (Invitrogen, PA3-981, SB249693) confirmed nuclear localization of the Myc-Bs protein. 24 h post-transfection, cells were harvested, combined into batches of ∼1×10^6^ cells/batch and processed. In brief, cells were treated with hypotonic buffer, lysed and centrifuged, and the nuclear protein fraction was obtained by extracting the pellet with high-salt buffer [20 mM HEPES (pH 7.9), 0.4 M NaCl, 1 mM EDTA, 1 mM EGTA, 1 mM DTT, 1 mM PMSF].

Oligonucleotides for EMSA probes were designed to carry 5′GpG dinucleotide overhangs after annealing (Table 1). To label EMSA probes, a fill-in reaction was run with annealed probes using Klenow exo^−^ enzyme (New England Biolabs) and P^32^-labeled dCTP. The labeled probes were purified on G-50 columns (GE Healthcare) and diluted to 50,000 cpm.

For each binding reaction, 3 µl of nuclear extract were mixed together with 1 µl of polydI/dC (Sigma, 1mg/ml) and 1 µl of 10× binding buffer [150 mM Hepes (pH 7.9), 10 mM EDTA, 5 mM DTT, 5% glycerol, 4 µg PolydI/dC] in the total volume of 10 µl. Such pre-assembled reactions received 1 µl of a labeled probe either alone or in combination with a 50× excess of non-labeled competitor probes, as indicated. After a 20 min incubation, reactions were loaded and run on 5% polyacrylamide gel. Probe signal was detected using X-ray film.

### Statistical analysis

A comprehensive summary on sample sizes, statistical methods and *P*-values can be found in Table S5.

## Supplementary Material

Supplementary information
