# Supplementary material for: Regulation of fiber-specific actin expression by the Drosophila SRF ortholog Blistered
Source: Development. 2019 Apr 4;146(7):dev164129. doi: 10.1242/dev.164129 (PMC6467476; doi:10.1242/dev.164129)
Supplement: Supplementary information [file develop-146-164129-s1.pdf]

Fig. S1. Involvement of *bs* in myogenesis is isoform-specific. A. Cross with the trachea-specific genetic driver *breathless-Gal4* (*btl-Gal4*) to functionally validate isoform-specific *bs* RNAi constructs. Knockdown efficiency was measured by determining the relative viability of non-*CyO* progeny compared to *CyO* progeny. If no lethality occurred, approximately 50% of progeny would be non-*CyO*. Control cross did not have RNAi transgenes, but retained the driver. Note that both RNAi constructs, targeting *bs*-RA/RC and -RB isoforms produced similar degree of lethality. **B.** Isoform-specific effects of *bs* knockdowns in adult muscles. RNAi was induced in all myoblasts using the *1151-Gal4* driver. The mean flight index was calculated for 55-75 flies in each group, as described in the Materials and Methods. For jump tests, the mean jumping distance for a group of 45-65 flies is shown. Only knockdown of *bs*-RA affected flying and jumping. All bars represent the average +/- standard deviation. t-test results comparing KD data with controls: **<0.01.

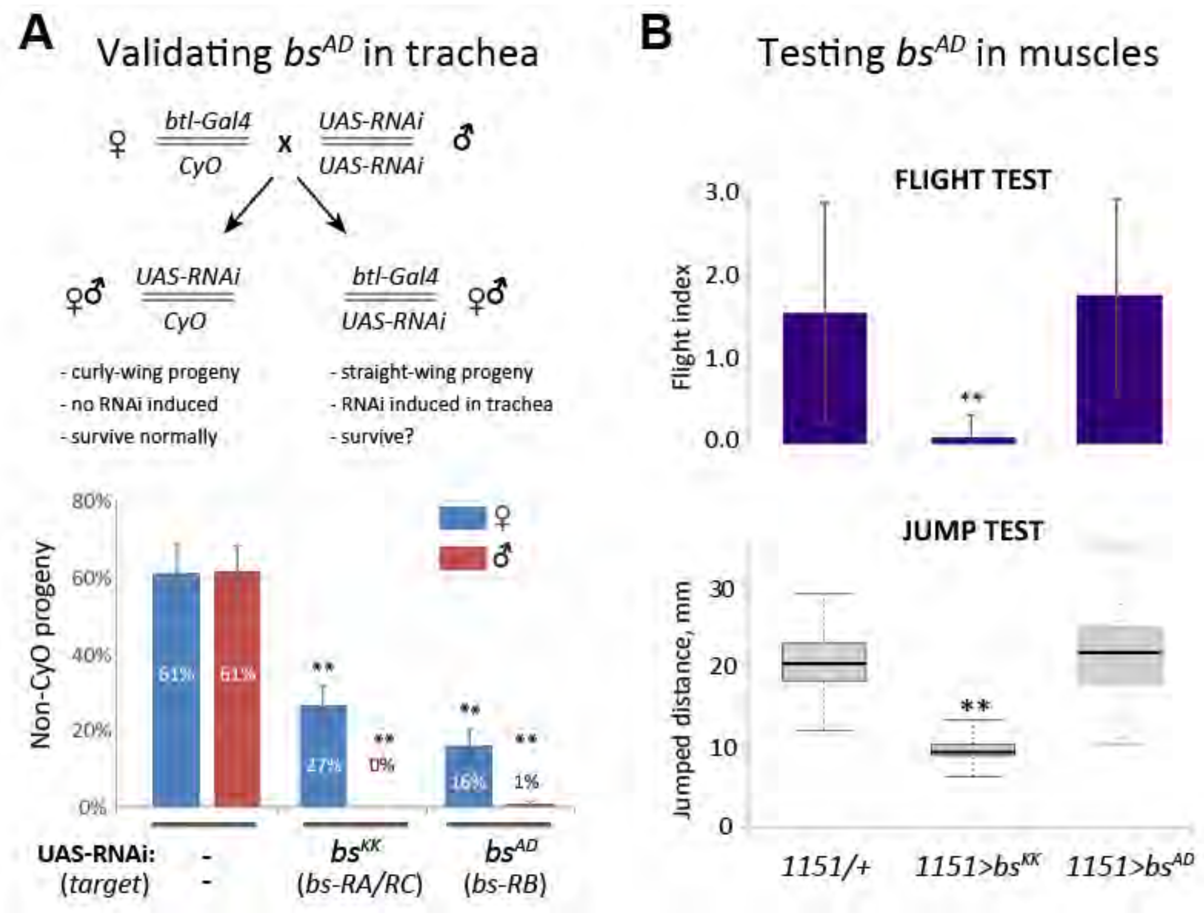

Development • Supplementary information

Fig. S2. Validation of *hth* knockdown in developing flight muscles produced by two genetic drivers. Hth protein (red), F-actin (green), and DNA (blue) in flight (IFM) and jump (TDT) muscles were detected by fluorescence confocal microscopy from 10-μm cryosections (maximum intensity projections were used). Quantification of the images (below) shows the median of Hth immunostaining intensity in IFM and TDT nuclei. TDT nuclei that naturally lack Hth were used to set the background level (dashed line). **A**. Control (*Mef2*^{TS}>+) and experimental (*Mef2TS>hth*^{RNAi}) pupae were moved to 29°C at 0h apf, to activate RNAi by temperature-sensitive driver *Mef2*^{TS}, and then subsequently analyzed at 48 h apf. **B**. Control (*fln*>+) and knockdown pupae (*fln>hth*^{RNAi}) were incubated at 25°C to induce *hth* KD at mid-developmental stage and analyzed as pharate adults near the end of pupal development (90+h apf). Arrowhead indicates Hth expressed in a tracheal cell outside of knockdown muscle.

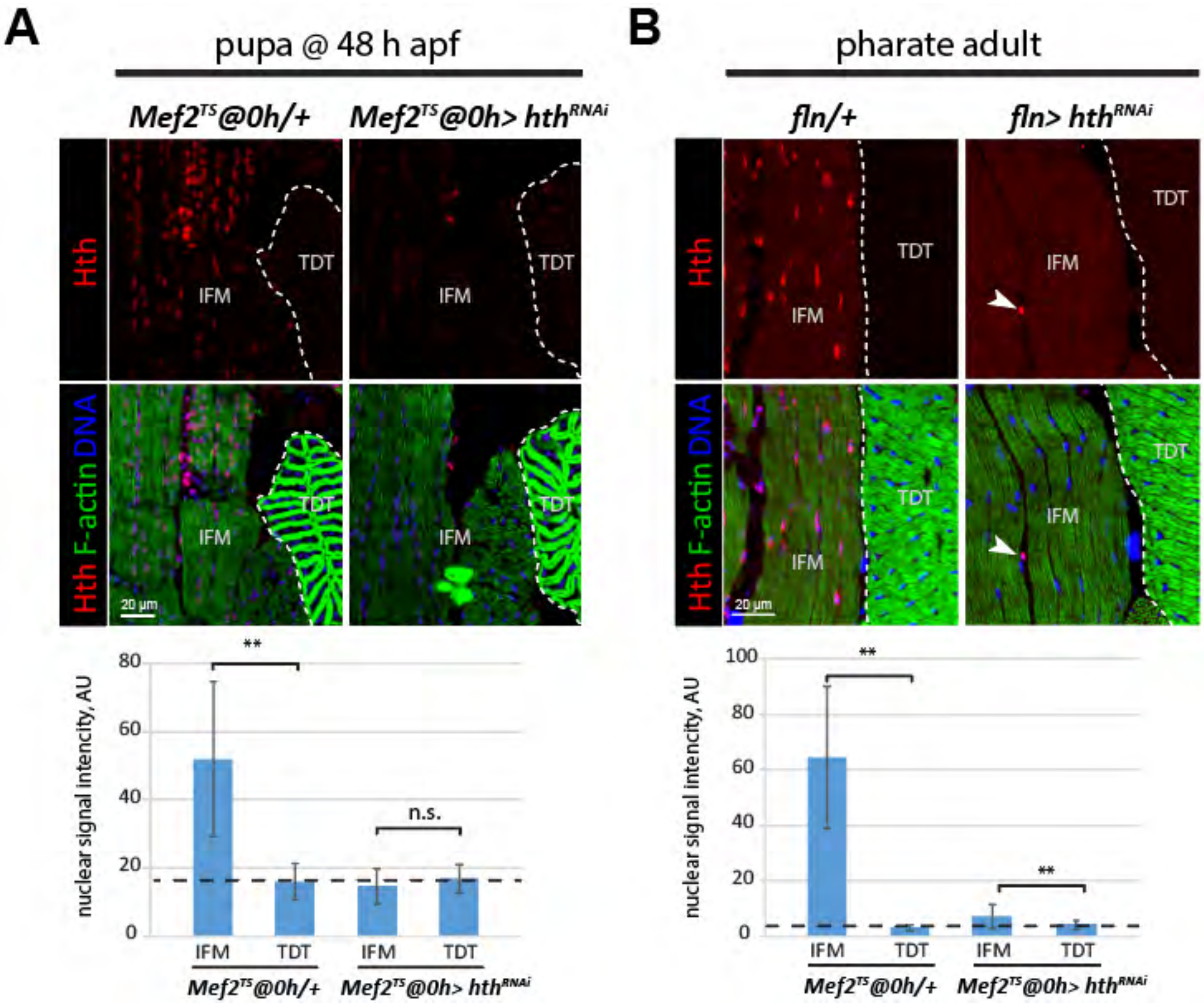

Fig. S3. Fixation protocol affects morphometric measurements of myofibrils. Flies were either fixed with 3.7% formaldehyde for 24 h at 4°C (pre-fixed) or left untreated (post-fixed), before cryosectioning and immunostaining as described in the Methods. **A.** A box plot of measured sarcomere lengths obtained from 48 h apf pupae. **B.** Box-plot distribution of myofibril diameters obtained from adult flies. Note that pre-fixation extends sarcomere lengths (A), but reduces myofibril diameters (B). Crosses, position of the mean; asterisks, t-test p-value <<0.01.

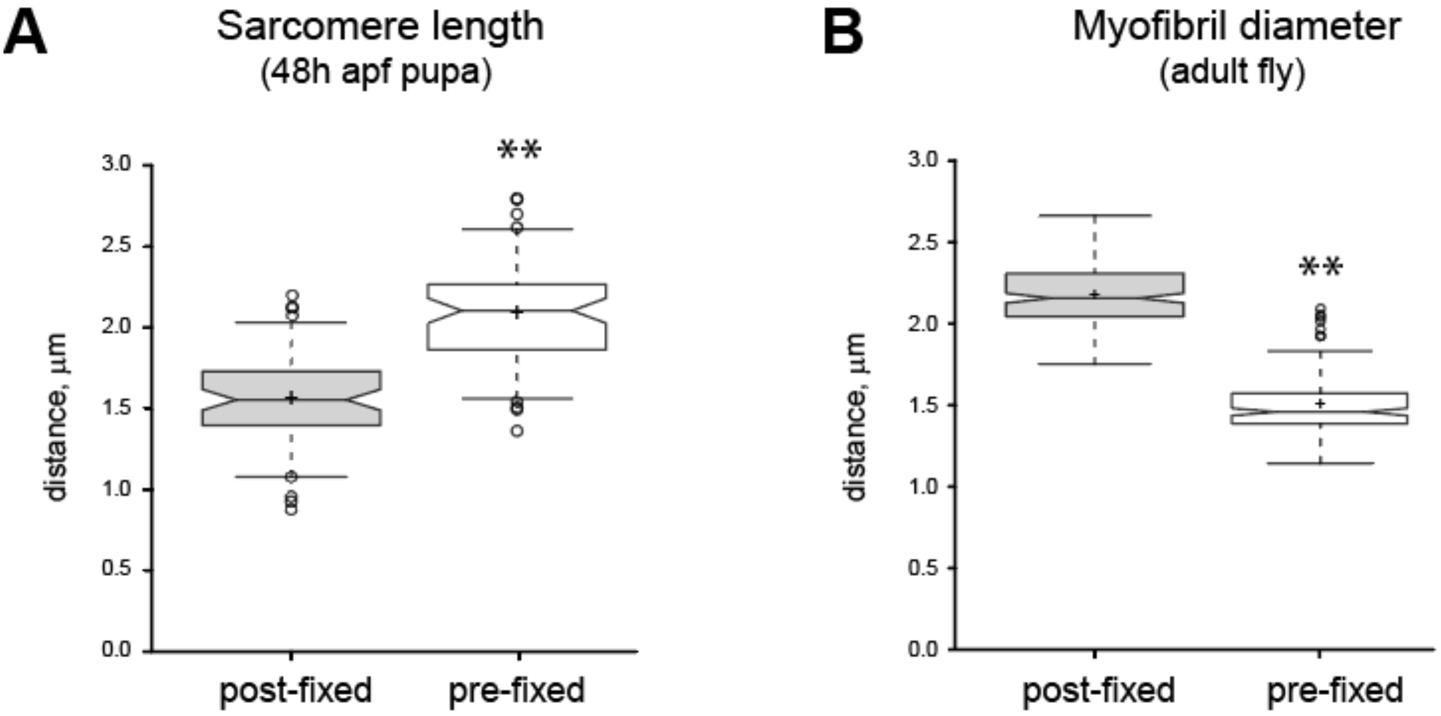

Table S1. Quantitative morphometric analysis of myofibrils in control and bs KD pupae and adult flies

Click here to Download Table S1

Table S2. Quantitative transcriptome data from control and bs knockdown flight muscles

Click here to Download Table S2

Table S3. The list of genes demonstrating significant changes in activity in the flight muscle in response to *bs* RNAi knockdown.

Flybase Gene ID	Expression, TPM*		Change in expression, log[2]	Known to respond to *Act88F*-null? #	Gene symbol	Notes
	control	bs KD				
FBgn0032381	399.5	2.1	-7.5		Mal-B1	enzyme, metabolic
FBgn0051202	9.0	0.2	-5.5		alpha-Man-Ic	enzyme
FBgn0015010	97.8	3.1	-5.0		Ag5r	-
FBgn0263762	384.2	36.6	-3.4		CG43679	-
FBgn0033521	100.0	10.1	-3.3		CG12896	redox homeostasis
FBgn0266455	78.2	12.8	-2.6		CG45080	-
FBgn0000047	33115.5	7471.5	-2.1		Act88F	myofiber component, actin, IFM-specific **identified as Bs target in this study**
FBgn0016926	87.2	23.1	-1.9		Pino	-
FBgn0031561	91.4	32.7	-1.5		IM33	immune response
FBgn0033518	98.3	38.5	-1.4		Prx2540-2	redox homeostasis
FBgn0039525	741.9	412.5	-0.8		CG5646	mitochondrial transporter
FBgn0032129	694.8	395.9	-0.8		jp	-
FBgn0040985	856.8	532.4	-0.7		CG6115	-
FBgn0010213	916.4	628.3	-0.5		Sod2	redox homeostasis
FBgn0261439	971.1	684.2	-0.5		SdhA	mitochondrial respiration
FBgn0002772	19455.8	25584.4	0.4		Mlc1	myofiber component, myosin light chain
FBgn0029971	897.3	1488.5	0.7		ND-MNLL	mitochondrial respiration
FBgn0266490	583.1	985.1	0.8		CG45089	-
FBgn0069923	959.3	1923.1	1.0		CG41128	-
FBgn0011726	76.1	168.5	1.1		tsr	actin binding
FBgn0031603	9.9	22.7	1.2		CG15432	-
FBgn0031057	9.6	22.1	1.2		Ubqn	proteasome component
FBgn0032147	68.3	160.5	1.2		IP3K1	kinase, regulatory
FBgn0000557	290.0	685.1	1.2		eEF1alpha2	translation machinery
FBgn0030433	71.0	168.6	1.2		mRpL49	translation machinery, mitochondrial
FBgn0038234	9.3	22.4	1.3		mRpL11	translation machinery, mitochondrial
FBgn0041205	8.3	20.3	1.3		key	protein adaptor, regulatory
FBgn0003714	86.3	214.0	1.3		tko	translation machinery, mitochondrial
FBgn0033608	237.3	600.8	1.3		CG13220	-
FBgn0040077	8.7	22.9	1.4		primo-1	phosphatase, regulatory
FBgn0035335	53.1	140.2	1.4		mRpL23	translation machinery, mitochondrial
FBgn0001223	404.4	1087.9	1.4	yes	Hsp22	molecular chaperone
FBgn0040899	75.5	207.2	1.5		CG17776	-
FBgn0039849	7.1	19.6	1.5		CG11334	enzyme, metabolic
FBgn0264694	8.4	23.2	1.5		mgr	molecular chaperone
FBgn0038674	9.7	27.3	1.5		CG14285	-
FBgn0033179	9.6	27.4	1.5		p47	proteasome component

Development • Supplementary information

FBgn0011476	74.8	213.6	1.5		l(3)neo43	putative mitochondrial product
FBgn0039765	55.4	163.8	1.6		mRpS18C	translation machinery, mitochondrial
FBgn0032247	5.9	17.9	1.6		CG5188	protease
FBgn0085284	7.0	21.3	1.6		Blos3	membrane transport
FBgn0029854	5.8	17.6	1.6		CG3566	putative mitochondrial product
FBgn0033529	6.4	19.7	1.6		CG17765	-
FBgn0013276	43.0	136.4	1.7	yes	Hsp70Ab	molecular chaperone
FBgn0050094	6.9	22.2	1.7		CG30094	-
FBgn0036580	8.7	28.5	1.7		PDCD-5	-
FBgn0030976	36.8	122.5	1.7		CG7378	phosphatase
FBgn0044511	97.6	325.6	1.7		mRpS21	translation machinery, mitochondrial
FBgn0031601	5.7	19.2	1.7		Dim1	regulation of mRNA splicing
FBgn0085452	222.9	751.0	1.8		CG34423	putative mitochondrial product
FBgn0259143	6.0	20.2	1.8		CG42258	putative membrane product
FBgn0033907	63.5	214.8	1.8		mRpS16	translation machinery, mitochondrial
FBgn0015299	6.1	20.6	1.8		Ssb-c31a	transcriptional cofactor
FBgn0050109	5.1	17.3	1.8		CG30109	putative mitochondrial product
FBgn0029134	45.2	154.1	1.8		Prosbeta5	proteasome component
FBgn0028515	4.9	17.2	1.8		EndoGI	DNA repair machinery
FBgn0033691	9.8	34.3	1.8		CG8860	transmembrane transporter, translocase
FBgn0040890	6.0	21.5	1.8		ksh	membrane transport
FBgn0261560	140.0	503.5	1.8	yes	Thor	translation machinery, regulatory
FBgn0035603	37.0	137.2	1.9		Pfdn4	molecular chaperone
FBgn0031822	6.4	24.0	1.9		Phf5a	mRNA splicing machinery
FBgn0036136	9.0	34.0	1.9	yes	Ufd1-like	proteasome component
FBgn0032240	5.3	19.9	1.9		CG17768	mRNA splicing machinery
FBgn0037199	7.7	29.2	1.9		CG11137	molecular chaperone
FBgn0015283	29.9	113.9	1.9		Rpn10	proteasome component
FBgn0033208	88.9	340.3	1.9		mRpL52	translation machinery, mitochondrial
FBgn0040389	33.9	131.5	2.0	yes	mRpL14	translation machinery, mitochondrial
FBgn0002645	4.7	18.3	2.0		Map205	structural, microtubule cytoskeleton
FBgn0035830	4.8	18.9	2.0		CG8209	-
FBgn0038426	60.1	234.8	2.0		mRpS33	translation machinery, mitochondrial
FBgn0026323	4.4	17.8	2.0		Tak1	kinase, regulatory
FBgn0062442	67.4	273.9	2.0		Cisd2	-
FBgn0002284	27.4	113.6	2.1		Prosbeta6	proteasome component
FBgn0028689	26.6	110.8	2.1	yes	Rpn6	proteasome component
FBgn0028693	34.8	145.6	2.1		Rpn12	proteasome component
FBgn0250746	34.1	142.7	2.1	yes	Prosbeta7	proteasome component
FBgn0083968	51.1	214.2	2.1		CG34132	mitochondrial transporter
FBgn0016697	30.3	128.2	2.1	yes	Prosalpha5	proteasome component
FBgn0033130	8.2	34.7	2.1		Tsp42Ei	-

Development • Supplementary information

FBgn0032200	33.3	142.4	2.1		CG5676	putative mitochondrial product
FBgn0040666	51.2	227.4	2.2		CG12848	translation machinery, mitochondrial
FBgn0038678	51.0	226.8	2.2	yes	mRpL55	translation machinery, mitochondrial
FBgn0020369	35.0	158.7	2.2	yes	Rpt6	proteasome component
FBgn0034033	5.9	27.6	2.2		CG8204	-
FBgn0033129	4.9	23.6	2.3		Tsp42Eh	membrane component
FBgn0037749	3.9	18.9	2.3		CG9471	-
FBgn0261394	21.5	105.3	2.3	yes	Prosalpha3	proteasome component
FBgn0031776	3.7	18.4	2.3		Pfdn1	molecular chaperone
FBgn0037788	36.0	176.9	2.3		CG3940	enzyme, putative
FBgn0032596	23.7	119.1	2.3	yes	Prosbeta4	proteasome component
FBgn0261014	21.0	106.5	2.3		TER94	proteasome component
FBgn0028691	21.5	110.6	2.4		Rpn9	proteasome component
FBgn0033810	3.2	16.5	2.4		CG4646	-
FBgn0050382	53.3	280.1	2.4	yes	CG30382	proteasome component
FBgn0033808	6.0	32.2	2.4		CG4627	-
FBgn0040907	36.2	200.6	2.5	yes	mRpL33	translation machinery, mitochondrial
FBgn0086134	22.2	127.5	2.5	yes	Prosalpha2	proteasome component
FBgn0028686	20.3	118.6	2.5		Rpt3	proteasome component
FBgn0261458	8.6	51.4	2.6		capt	actin binding
FBgn0003996	64.1	408.8	2.7		w	transmembrane transporter
FBgn0266666	27.2	175.6	2.7	yes	Sem1	proteasome component
FBgn0031992	8.2	53.4	2.7		CG8498	-
FBgn0037378	2.9	19.3	2.7		CG2046	proteasome component
FBgn0032216	8.5	56.5	2.7		Usp14	proteasome component
FBgn0028690	10.0	66.5	2.7	yes	Rpn5	proteasome component
FBgn0038652	5.2	38.1	2.9		CG7720	transmembrane transporter
FBgn0005536	16.6	125.7	2.9	yes	Mbs	non-muscle myosin regulatory subunit
FBgn0031974	6.2	48.6	3.0		CG12560	histone acetylase
FBgn0031907	2.1	16.6	3.0		CG5171	enzyme, biosynthetic
FBgn0033781	2.4	20.3	3.1		CG13319	proteasome component
FBgn0040493	2.1	17.9	3.1		grsm	protease
FBgn0250843	6.8	58.1	3.1	yes	Prosalpha6	proteasome component
FBgn0013278	12.6	111.0	3.1		Hsp70Bb	molecular chaperone
FBgn0264389	1.8	16.7	3.2		opm	transmembrane transporter
FBgn0011296	1.3	13.3	3.3		l(2)efl	molecular chaperone
FBgn0038577	2.1	20.8	3.3		CG12321	proteasome component
FBgn0020907	2.5	25.3	3.3		Scp2	-
FBgn0039637	1.8	18.5	3.3		Ctl2	-
FBgn0013348	2.0	21.3	3.4	yes	TpnC41C	myofiber component, troponin C, non-IFM specific
FBgn0085736	1.7	19.5	3.5		CG40472	putative mitochondrial product

Development • Supplementary information

FBgn0010397	1.4	16.6	3.6		LamC	structural, nuclear envelope
FBgn0000044	2.1	31.5	3.9		Act57B	myofiber component, actin, non-IFM specific
FBgn0033926	12.1	190.6	4.0	yes	Arc1	regulatory
FBgn0042173	5.2	83.1	4.0		CG18853	DNA repair machinery
FBgn0037819	15.0	241.9	4.0		CG14688	enzyme
FBgn0024315	1.2	22.2	4.2		Picot	transmembrane transporter
FBgn0033928	0.6	18.8	4.9		Arc2	-
FBgn0039452	0.4	12.5	5.1		CG14245	chitin binding
FBgn0030999	0.4	15.1	5.2		Mur18B	extracellular
FBgn0264753	0.4	14.3	5.3		Rgk1	regulatory
FBgn0036656	3.0	128.6	5.4		CG13026	-
FBgn0026438	0.0	10.8	9.0		Eaat2	transmembrane transporter
FBgn0033788	0.0	10.9	10.1		CG13323	-
FBgn0051087	0.0	53.0	10.2		CG31087	-
FBgn0035343	0.0	11.9	10.2		CG16762	-
FBgn0052656	0.0	20.1	11.0		Muc11A	extracellular

* Transcripts per million, as determined by the mapping algorithm (1)

Genes previously reported to upregulate expression in IFMs lacking muscle-specific actin, as reported in (2)

1. Patro R, Duggal G, Love M, Irizarry R, & Kingsford C (2016) Salmon provides accurate, fast, and bias-aware transcript expression estimates using dual-phase inference. bioRxiv.
2. Madan A*, et al.* (2017) Transcriptome analysis of IFM-specific actin and myosin nulls in Drosophila melanogaster unravels lesion-specific expression blueprints across muscle mutations. *Gene* 631:16-28.

Development • Supplementary information

Table S4. Gene ontology analysis for the genes responding to bs knockdown in flight muscles

Analysis Type:	PANTHER Overrepresentation Test (Released 20171205)						
Annotation Version and Release Date:	GO Ontology database Released 2018-07-03						
Analyzed List:	all genes upregulated in bs KD						
Reference List:	Drosophila melanogaster (all genes in database)						
Test Type:	FISHER						
GO biological process complete	Drosophila	UP in bs KD	UP in bs KD	UP in bs KD	UP in bs KD	UP in bs KD	UP in bs KD IFMs (FDR)
cellular process (GO:0009987)	6613	93	58.16	+	1.6	1.68E-10	6.71E-08
cellular metabolic process (GO:0044237)	3835	58	33.73	+	1.72	3.40E-06	6.29E-04
nitrogen compound metabolic process (GO:0006807)	3729	54	32.8	+	1.65	5.04E-05	7.81E-03
macromolecule metabolic process (GO:0043170)	3216	52	28.29	+	1.84	2.37E-06	4.50E-04
organonitrogen compound metabolic process (GO:1901564)	2787	51	24.51	+	2.08	4.86E-08	1.37E-05
cellular macromolecule metabolic process (GO:0044260)	2179	48	19.17	+	2.5	3.36E-10	1.28E-07
protein metabolic process (GO:0019538)	2113	47	18.58	+	2.53	4.15E-10	1.50E-07
cellular protein metabolic process (GO:0044267)	1626	46	14.3	+	3.22	1.88E-13	8.40E-11
catabolic process (GO:0009056)	835	38	7.34	+	5.17	2.66E-17	1.44E-14
proteolysis (GO:0006508)	824	37	7.25	+	5.11	1.20E-16	6.05E-14
cellular catabolic process (GO:0044248)	758	38	6.67	+	5.7	1.18E-18	7.48E-16
organic substance catabolic process (GO:1901575)	745	37	6.55	+	5.65	5.13E-18	3.00E-15
cellular response to stress (GO:0033554)	526	16	4.63	+	3.46	1.89E-05	3.11E-03
organonitrogen compound catabolic process (GO:1901565)	475	36	4.18	+	8.62	3.04E-23	2.31E-20
macromolecule catabolic process (GO:0009057)	466	35	4.1	+	8.54	1.85E-22	1.28E-19
cellular macromolecule catabolic process (GO:0044265)	408	35	3.59	+	9.75	3.05E-24	2.57E-21
protein catabolic process (GO:0030163)	316	35	2.78	+	12.59	1.07E-27	1.02E-24
cellular protein catabolic process (GO:0044257)	307	35	2.7	+	12.96	4.36E-28	4.73E-25
proteolysis involved in cellular protein catabolic process (GO:0051603)	306	35	2.69	+	13	3.94E-28	4.98E-25
modification-dependent macromolecule catabolic process (GO:0043632)	286	35	2.52	+	13.91	4.78E-29	7.26E-26
modification-dependent protein catabolic process (GO:0019941)	280	35	2.46	+	14.21	2.47E-29	4.68E-26
ubiquitin-dependent protein catabolic process (GO:0006511)	259	35	2.28	+	15.36	2.17E-30	5.49E-27
proteasomal protein catabolic process (GO:0010498)	184	33	1.62	+	20.39	3.26E-32	1.24E-28
proteasome-mediated ubiquitin-dependent protein catabolic process (GO:0043161)	174	33	1.53	+	21.56	6.41E-33	4.87E-29
protein folding (GO:0006457)	142	8	1.25	+	6.41	4.96E-05	7.84E-03
response to heat (GO:0009408)	89	7	0.78	+	8.94	2.02E-05	3.26E-03
positive regulation of protein complex assembly (GO:0031334)	53	6	0.47	+	12.87	1.20E-05	2.02E-03
chaperone-mediated protein folding (GO:0061077)	44	7	0.39	+	18.09	2.71E-07	5.71E-05
response to topologically incorrect protein (GO:0035966)	44	6	0.39	+	15.5	4.50E-06	7.77E-04
cellular response to heat (GO:0034605)	44	6	0.39	+	15.5	4.50E-06	7.95E-04
cellular response to topologically incorrect protein (GO:0035967)	43	6	0.38	+	15.86	3.99E-06	7.22E-04
response to unfolded protein (GO:0006986)	36	6	0.32	+	18.95	1.57E-06	3.06E-04
'de novo' protein folding (GO:0006458)	35	7	0.31	+	22.74	6.74E-08	1.83E-05

Development • Supplementary information

cellular response to unfolded protein (GO:0034620)	35	6	0.31	+	19.49	1.36E-06	2.71E-04
ERAD pathway (GO:0036503)	30	6	0.26	+	22.74	6.10E-07	1.25E-04
proteasomal ubiquitin-independent protein catabolic process (GO:0010499)	25	12	0.22	+	54.57	1.70E-16	8.08E-14
protein refolding (GO:0042026)	25	7	0.22	+	31.83	9.03E-09	2.86E-06
regulation of DNA-templated transcription initiation (GO:2000142)	24	6	0.21	+	28.42	1.94E-07	4.21E-05
ubiquitin-dependent ERAD pathway (GO:0030433)	24	6	0.21	+	28.42	1.94E-07	4.33E-05
regulation of transcription initiation from RNA polymerase II promoter (GO:0060260)	24	6	0.21	+	28.42	1.94E-07	4.46E-05
'de novo' posttranslational protein folding (GO:0051084)	21	6	0.18	+	32.48	9.88E-08	2.42E-05
chaperone cofactor-dependent protein refolding (GO:0051085)	21	6	0.18	+	32.48	9.88E-08	2.50E-05
proteasome assembly (GO:0043248)	15	8	0.13	+	60.64	1.16E-11	4.90E-09
positive regulation of DNA-templated transcription initiation (GO:2000144)	15	6	0.13	+	45.48	1.89E-08	5.52E-06
positive regulation of transcription initiation from RNA polymerase II promoter (GO:006	15	6	0.13	+	45.48	1.89E-08	5.74E-06
regulation of RNA polymerase II transcriptional preinitiation complex assembly (GO:004	10	6	0.09	+	68.22	2.89E-09	9.54E-07
polytene chromosome puffing (GO:0035079)	10	5	0.09	+	56.85	1.30E-07	3.08E-05
positive regulation of RNA polymerase II transcriptional preinitiation complex assembly	9	6	0.08	+	75.8	1.82E-09	6.28E-07
heat shock-mediated polytene chromosome puffing (GO:0035080)	9	5	0.08	+	63.16	8.72E-08	2.28E-05

Development • Supplementary information

Table S5. summary of statistical analyses.

	Parameter, comparative pair	Sample description	Comparative group sizes	Statistical Method	p-value (statistically significant in bold font)
Fig 1 D	*bs* knockdown quantification by qPCR in IFM, control vs bs KD	cDNA preps from dissected muscles, prepared from 3-5 flies	3 vs 3	Welch t-test	**7.3E-6**
	bs knockdown quantification by qPCR in TDT, control vs bs KD		3 vs 3	Welch t-test	**6.6E-4**
Fig 2 B	Myofibril thickness, 48h apf, control vs bs KD	Flies, each representing a median of 20-100 individual myofibril measurements	5 vs 6	Student t-test	0.90
	Myofibril thickness, young adult, control vs bs KD		7 vs 7	Welch t-test	**1.1E-08**
	Myofibril thickness, young adult, control vs rescue		7 vs 6	Student t-test	**3.9E-08**
	Myofibril thickness, young adult, bs KD vs rescue		7 vs 6	Welch t-test	**2.7E-08**
	Sarcomere length, 48h apf, control vs bs KD		5 vs 6	Student t-test	0.20
	Sarcomere length, young adult, control vs bs KD		7 vs 7	Welch t-test	**0.01**
	Sarcomere length, young adult, control vs rescue		7 vs 4	Welch t-test	0.17
	Sarcomere length, young adult, control vs rescue		7 vs 4	Student t-test	0.30
Fig 3 D	*bs*-RA/RC expression quantification by qPCR, IFM vs TDT	cDNA preps from dissected muscles, from 3-5 flies	3 vs 3	Student t-test	**0.01**
	bs-RB expression quantification by qPCR, IFM vs TDT		3 vs 3	Student t-test	**0.0037**
Fig 5 A	Actin protein expression quantification in IFM lysates	Protein lysate from dissected muscles obtained from 8 flies	4 vs 4	Welch t-test	**0.009**
	Actin protein expression quantification in TDT lysates	Protein lysate from dissected muscles, obtained from 8 flies	4 vs 4	Welch t-test	0.81
Fig 5 B	*Act88F* gene expression quantification by qPCR, control vs bs KD	cDNA prep from 4 flies	3 vs 3	Welch t-test	**0.008**
Fig 5 C	Reporter activity quantification, control vs bs KD	Lysate prepared from 3 flies	3 vs 3	Student t-test	**0.004**
Fig 6 D	*Act88-FL* reporter activity quantification, control vs bs KD	Lysate prepared from 3 flies	3 vs 3	Welch t-test	**6.48E-04**
	Act88-AB reporter activity quantification, control vs bs KD	Lysate prepared from 3 flies	3 vs 3	Student t-test	0.05
Fig S1 A	Female fly survival to adulthood, control vs UAS-bs^{KK}	Batches of flies (70-200 flies) produced by independent crosses	3 vs 3	Student t-test	0.006
	Female fly survival to adulthood, control vs UAS-bs^{AD}		3 vs 3	Student t-test	0.002

Development • Supplementary information

	Male fly survival to adulthood, control vs UAS-bs^{KK}		3 vs 3	Student t-test	2.12E-04
	Male fly survival to adulthood, control vs UAS-bs^{AD}		3 vs 3	Student t-test	2.27E-04
Fig S1 B	Flight index, control vs UAS-bs^{KK}	Individual flies (both sexes)	63 vs 76	Welch t-test	3.17E-30
	Flight index, control vs UAS-bs^{AD}		63 vs 63	Student t-test	0.38
	Jumping distance, control vs UAS-bs^{KK}		56 vs 63	Welch t-test	3.17E-30
	Jumping ability, control vs UAS-bs^{AD}		56 vs 46	Student t-test	0.37
Fig S2 A	Fluorescence signal intensity, IFM vs TDT, control	1 nucleus	53 vs 47	Welch t-test	**6.29E-19**
	Fluorescence signal intensity, IFM vs TDT, hth KD		50 vs 45	Student t-test	0.3
Fig S2 B	Fluorescence signal intensity, IFM vs TDT, control	1 nucleus	25 vs 25	Welch t-test	**1.11E-11**
	Fluorescence signal intensity, IFM vs TDT, hth KD		25 vs 25	Student t-test	**2.49E-17**
Fig S3 A	Sarcomere length in IFMs, 48h apf, post-fixed vs pre-fixed samples	1 sarcomere	75 vs 75	Student t-test	**3.15E-19**
Fig S3 B	Myofibril diameter, adult IFMs, post-fixed vs pre-fixed samples	1 myofibril	195 vs 169	Student t-test	**1.44E-120**

Development • Supplementary information
